# Challenges and Strategies in Hydrogel-Based Cartilage Regeneration

**DOI:** 10.3390/gels12050350

**Published:** 2026-04-22

**Authors:** Carola Cavallo, Emanuela Amore, Sara Carpentieri, Livia Roseti

**Affiliations:** Laboratorio RAMSES, Dipartimento Rizzoli Research & Innovation Technology, IRCCS Istituto Ortopedico Rizzoli, 40136 Bologna, Italy; carola.cavallo@ior.it (C.C.); sara.carpentieri@ior.it (S.C.); livia.roseti@ior.it (L.R.)

**Keywords:** hydrogels, articular cartilage, hyaline-like tissue regeneration, fibrosis, biocompatibility, rejection

## Abstract

The increase in older adults and active lifestyles has made chondral and osteochondral lesions common in the population, making them one of the central challenges in orthopedics. Although hydrogel-based regenerative medicine offers an encouraging therapeutic option for these lesions, important obstacles still prevent these therapies from reaching the clinic. In view of these factors, we adopted a risk-based approach for this review, in line with the current legislative requirements in clinical translation and clinical trials. We identified the factors that could undermine patient safety or lead to poor outcomes. Then, we outlined solutions to remedy these problems that integrate hydrogel technology, clinical/pharmaceutical/surgical protocols, and post-operative follow-up. Upcoming studies should give priority to the development of hydrogel scaffolds modified to mimic cartilage’s mechanical and physicochemical properties, together with patient-specific features. Other crucial characteristics are host-tissue integration, long-lasting cartilage tissue regeneration, and a positive outcome. In parallel, to scale complex and costly innovations, efforts should focus on a harmonized, simplified legislative landscape, optimized standards, and established follow-up protocols. Getting through this “valley of death” between research and innovation is strategic for reaching the clinics and the largest number of patients.

## 1. Introduction

The increase in older adults and active lifestyles has made chondral and osteochondral lesions, especially of the knee, common in the population. However, these injuries, caused by trauma, repetitive movements, or degenerative disease, are still one of the crucial issues in orthopedics [1,2].

Articular hyaline cartilage is a highly specialized joint tissue that facilitates the transmission of loads with low friction. The progenitor cells’ infiltration and repair processes are limited due to the absence of vascular and lymphatic vessels in this tissue [3]. Moreover, when the damage extends to the subchondral bone, progenitors from the bone marrow process act by producing a fibrous tissue that could be functionally active for a brief period but does not present the mechanical and strength characteristics of hyaline cartilage.

There is no direct correlation between articular cartilage lesions and symptoms or disability. Nevertheless, cartilage damage, if left untreated, may worsen, causing pain, impaired locomotion, inflammation, and fibrosis, typical traits of Osteoarthrosis, where an inflamed joint environment further negatively impacts the repair process [4].

The types of cartilage injury and degeneration vary in size, shape, depth, location, patient age, and joint situation, making a range of treatments necessary.

The scenario of the management of articular cartilage defects is described in Table 1. Such a scenario starts with palliative care, including non-pharmacological approaches such as physical therapy and exercises, as well as pharmacological therapies, including systemic drug administration, topical medications, and intra-articular injections. Those clinical approaches focus on symptom relief. Surgical reparative approaches like abrasions, drilling, and microfracture, although suitable for young, active patients with severe symptoms or full-thickness injuries, can result in fibrocartilage formation [5].

Regenerative treatments should offer an alternative to conventional methods by replacing damaged cartilage with new tissue of similar characteristics, restoring joint function, and reducing pain [6]. They combine different technologies to develop a variety of therapeutic solutions: From the initial Autologous Chondrocyte Implantation (ACI) using ex vivo manipulated cells, through tissue engineering combining ex vivo manipulated chondrocytes or Mesenchymal Stem Cells (MSCs) with scaffolds and chemical or mechanical signals [7].

Beyond this, other autologous alternatives used are minimally manipulated cells, avoiding the ex vivo phase, such as BMAC (Bone Marrow Aspirate Concentrate) and PRP (Platelet-Rich Plasma), that can be administered in combination to strengthen regenerative effects [8].

In parallel with cell-based strategies, cell-free approaches use scaffolds, acellular matrices, or exosomes. These are extracellular vesicles carrying a signaling cargo representing the treatment, instead of the cells themselves [9].

Different from the above-described methods, which are already clinical realities or in an advanced investigational phase, induced Pluripotent Stem Cells (iPSCs) remain in the pre-clinical phase due to safety concerns [10].

Gene Therapy is an advanced technology that acts by introducing engineered genetic material into the target cells, but its application in cartilage treatment is still in the early stages [11].

**Table 1:** Scenario of the management of articular cartilage defects.

Approach	Therapy Type	Pros	Cons	Refs.
Palliative	Physiotherapy/exercise: muscular strengthening and joint mobilization	Non-invasive, low cost, improves stability, exerts additional effects on inflammation and muscle hypertrophy	Does not repair tissue damage; temporary relief; efficacy moderated by the patient’s age and comorbidities, the intervention time, and the follow-up duration	[12]
	Drug administration: systemic use of non-steroidal anti-inflammatory drugs (NSAIDs) or analgesics	Easy to administer, rapid pain control	Side effects (gastrointestinal, renal). Does not stop degeneration; long-term use could accelerate the progression to total knee replacement by markedly exacerbating symptoms	[13]
	Intra-articular injections of analgesics, and viscosupplementation with hyaluronic acid, glucosamine, and chondroitin sulfate	Minimally invasive	Risk of infection; temporary effect	[14]
	Use of orthotics: braces or specialized footwear	Corrects alignment, reduces load on the lesion	Patient compliance; skin irritation; bulky	[15]
	Arthroscopic debridement/chondral shaving/chondroplasty: removal of all the debris deriving from the damaged joint: inflammatory cells, unstable chondral flaps, osteophytes, superfluous synovia, degenerated meniscus, and torn ligament fragments	Minimally invasive; rapid improvement of pain symptoms	Short-term relief; does not regenerate cartilage; may lead to further thinning of cartilage	[16]
	Joint lavage: rinsing of the joint with physiological fluids to wash out degradation products	Flushes out inflammatory cytokines and debris, minimally invasive	Short-term effect	[17]
Reparative	Bone marrow stimulation: abrasions, drilling, and microfracture: enclose a phase consisting of the subchondral bone penetration, thus inducing bleeding, and the migration of bone marrow MSCs to the site of injury, along with blood clot formation	Technically easy, low-cost, one-stage procedure; excellent short-term clinical outcomes have been demonstrated	The resulting repair tissue is composed of fibrocartilage. The clinical durability of the repair tissue has shown a functional decline with further follow-up	[18]
Restorative/reconstructive	Knee arthroplasty: total or partial joint replacement prosthetic implants	Decrease pain and improve mobility in people suffering from end-stage lesions	Invasive procedure, risk of stiffness, instability, aseptic loosening, infection, prosthesis failure, and malalignment	[19]
Regenerative	Osteochondral grafting: autologous osteochondral transplantation (AOT) or mosaicplasty, harvest of plugs from non-weight-bearing areas	Use of autologous hyaline cartilage; immediate availability; no risk of disease transmission	Limited graft supply; donor site morbidity; graft failure due to marginal chondrocyte death	[20]
	Osteochondral grafting: Allogeneic (allograft transplantation): transplantation of donor grafts	No donor site morbidity; treats large defects	Graft availability, possible disease transmission; short cell viability; biomechanical integrity of the donor graft	
	Cell therapy implantation of a suspension of previously GMP *-expanded chondrocytes: Autologous Chondrocyte Implantation (ACI) (1st generation ACI)	Uses autologous cells; long-term results	Two surgeries; periosteal hypertrophy risk; high cost of expansion; regulatory hurdles; safety of media supplements; de-differentiation of chondrocytes in monolayer cultures (switch to a fibroblastic phenotype)	[7,21]
	Cell therapy: implantation of a suspension of previously GMP *-expanded chondrocytes under a sutured, inert collagen membrane, (C-ACI), (2nd generation ACI)	Avoid periosteal flap harvesting as for ACI 1st generation	High cost; requires two surgical procedures	[21]
	Cell therapy: implantation of a suspension of previously GMP * expanded mesenchymal stem cells MSCs)/progenitors	High number of cells; multipotent potential; immunomodulatory effects; immune privilege; scalability	Risk of hypertrophy/ossification; high cost of expansion; regulatory hurdles; safety of media supplements	[22,23]
	Cell therapy: implantation of a suspension of previously GMP * expanded Adipose tissue(-Derived) Stem Cells (ASCs or ADSCs)	Abundant tissue source; high proliferative potential; easier to harvest than BM; immunomodulatory properties; low immunogenicity:	High cost of expansion; regulatory hurdles; safety of media supplements	[24]
	Cell therapy: implantation of a suspension of previously GMP * expanded Synovial Tissue-Derived Stem Cells (SDSCs)	High chondrogenic potential; tissue-specific lineage (shares niche with cartilage)		[25]
	Cell therapy: implantation of a suspension of previously GMP * expanded allogeneic Umbilical Cord Mesenchymal Stem Cells (UCMSCs)s	High proliferative capacity	Ethical/regulatory hurdles; long-term safety issues; high cost of expansion	[26]
	Cell therapy: implantation of a suspension of previously GMP * expanded induced Pluripotent Stem Cells (iPSCs)	Theoretically unlimited supply and scalability; reduced immunogenicity; genetic customization; high-quality control	Still in the pre-clinical stage (stringent safety controls required); tumorigenic risk; genetic and epigenetic instability; high cost and complexity of expansion; regulatory hurdles	[10,27]
	Cell-free therapy: scaffold alone used to guide tissue growth. Can be combined with bone marrow stimulation (e.g., microfracture)	One-stage; no cell expansion costs; provides structural support for endogenous cell recruitment; when in combination with bone marrow stimulation, the scaffold acts to recruit native MScs from the patient’s own bone marrow	Requires surrounding cells for migration; risk of inhomogeneous cell infiltration	[28]
	Cell-free therapy: acellular cartilage matrix (ACM), allogenic, or xenogenic	Mimics natural ECM; promotes host integration	Risk of immunogenicity	[29]
	Cell-free therapy: extracellular vesicles/exosomes from MSCs or chondrocytes	Low immunogenicity; no risk of cell transformation	Still in experimental stages, dosage is not standardized	[9]
	Tissue engineering: implantation of a construct composed of previously GMP * expanded chondrocytes seeded onto or into a 3D scaffold (3rd generation ACI)	The 3D scaffold improves cell retention and supports phenotype stability	Cost of two procedures; demanding GMP compliance	[21]
	Tissue engineering: implantation of a construct composed of GMP * expanded MSCs seeded onto or into a 3D scaffold	The 3D scaffold improves cell retention; possibility to use three-layered scaffolds and growth factors stimulating MSCs differentiation towards the chondrogenic or the osteogenic lineage (osteochondral regeneration)		[30]
	Scaffold-free therapy: Implantation of High-Density Autologous Chondrocyte (HD-ACI)	Promotes robust cell-to-cell signaling; minimizes dedifferentiation of chondrocytes	Mimics natural tissue density; no synthetic material risks	[31]
	Scaffold-free therapy: implants of spherical aggregates of GMP * expanded chondrocytes with a self-synthesized extracellular matrix (ECM), for instance Chondrocyte-based spheroids (third-generation ACI)	Uniform cell distribution; avoids scaffold-induced inflammation	Higher cost due to specialized culture techniques	[21]
	Synthetic growth factors: Transforming Growth Factor-beta (TGF-β), Kartogenin, Bone Morphogenetic Protein-2 (BMP-2), Insulin-like Growth Factor-1 (IGF-1)	Potentiates natural healing; minimally invasive (if injected)	Short half-life; side effects	[32]
	Biologic growth factors: Platelet-rich plasma (PRP) + hyaluronic acid, multiple injections	Autologous cocktails; minimally invasive application	High inter-patient variability; mechanisms not fully elucidated	[33]
	Concentrates: bone marrow Aspirate Concentrate (BMAC)	One-stage procedure; cocktail of cells/growth factors	Variability between patients; lower cell concentration than cultured cells; lack of standardization	[34]
	Concentrates: stromal vascular fraction (SVF) from adipose tissue			[35]
	Concentrates and biologic growth factors: combination of BMAC and PRP	Synergistic effect of BMAC and PRP	Increased procedure time; lack of standardized mixing ratios	[8]
	Concentrated associated with scaffolds and biologic growth factors: Combination of PRP and BMAC on a scaffold (for instance, collagen)	Scaffold provides mechanical stability and localized concentration of bioactive factors	Variability between patients; lower cell concentration than cultured cells; lack of standardization	[35]
	Concentrates associated with scaffolds: SVF on a biomaterial			[21]
	Minimally manipulated cells: cartilage chondrons mixed with allogeneic MSCs (4th-generation ACI, one-stage ACI)	One-stage procedure	Limited cell numbers	[36]
	Minimally manipulated cells: morselized cartilage implantation mixed with fibrin sealant or PRP (4th-generation ACI, one-stage ACI)	Technically easy; low cost; utilizes hyaline cartilage cues	Variable tissue quality; limited to smaller defects	[21,37]
	Minimally manipulated cells: juvenile allogeneic cartilage fragments and micromosaic plastic (4th-generation ACI, one-stage ACI, off-the-shelf)	One-stage; immediate hyaline matrix cues; no donor morbidity	Risk of immunogenicity	[21]
	Gene Therapy: 1. viral or non-viral local delivery of genes encoding growth factors, transcription factors, or anti-inflammatory proteins and non-coding RNAs through viral and non-viral vectors: 2. In vivo or direct delivery of the target gene to the lesion site by surgical incision or local injection 3. Ex vivo, GMP * transfection or infection of the cells, followed by delivery of the cells to the target tissue (ex vivo) 4. Biomaterials as gene delivery carriers	Long-term expression of therapeutic factors	Safety concerns (viral vectors); potential for uncontrolled over-expression	[11]
	Gene editing, CRISPR/Cas9 technology: precise ex vivo or in vivo modification of inflammatory or hypertrophic genes within cells used for repair	Precise modification of specific genes	Off-target effects; regulatory issues	[38]

* GMP = Good Manufacturing Practice.

Among the available biomaterials, hydrogels represent the solution of choice for tissue regeneration, thanks to their easy synthesis and highly tunable physical and mechanical properties [39]. Depending on the cartilage strategy applied, hydrogels can be utilized in (i) tissue engineering (GMP cells + scaffold), where they represent a 3D carrier for implanted cells; moreover, hydrogels are bio-inks used in 3D bioprinting. They allow for the creation of patient-specific, anatomically perfect implants that match the defect geometry; (ii) minimally manipulated cells/growth factors since they are often used as a stabilizer for BMAC or PRP, avoiding dispersion in the joint; (iii) cell-free therapy (scaffold alone) by acting as an acellular matrix that functions as a temporary structure to recruit the body’s own cells to the injury site; (iv) extracellular vesicles/growth factors acting as a sustained- and controlled-release reservoir [39].

### 1.1. Rationale and Aim

Hydrogels are considered a promising biomaterial advancement for cartilage regeneration due to their tunability to (i) address geometric adaptability; for example, injectable hydrogels are liquids that turn into solids (gelation) inside the joint, contouring to the irregular shape of a lesion, and providing lateral contact; (ii) mimic the high hydration nature of cartilage, creating a physiologic-like environment favoring cell viability and ECM synthesis; (iii) be designed as “Smart” and/or “Self-Healing” materials. The first are responsive hydrogels that respond to various physicochemical changes in the environment; the second can automatically heal and restore damage, avoiding premature failure caused by mechanical damage after implantation [40].

This review adopts a patient-risk perspective on the use of hydrogel materials for cartilage regeneration. This approach is becoming mandatory, given the growing demand for a risk-based approach in clinical translation and clinical trials [41].

The aim of this review is to focus on challenges that hamper the therapeutic effectiveness of hydrogel-based treatments, including patient safety and the quality of the hydrogel-based constructs implanted or injected. To reduce complication risks or compromised outcomes, the focus then moves to risk reduction strategies, including material design, regenerative techniques, patient monitoring, the legal panorama, and manufacturing.

### 1.2. Search Strategy

The selection process was conducted between 1 January and 15 February 2026, involving filtering peer-reviewed publications in English across PubMed (MEDLINE) and Scopus databases. We used the search string: MeSH (“cartilage regeneration/repair and hydrogels” [Mesh]) AND “Advantages/Pros and Disadvantages/Contras” [Mesh] AND “mitigation action/strategies” [Mesh].

For the search, we chose the 1990–2026 period to capture the evolution of cartilage regeneration from the landmark 1994 study by Brittberg et al. in the New England Journal of Medicine, which introduced Autologous Chondrocyte Implantation (ACI) for knee cartilage defects [7]. We excluded the gray literature, including conference abstracts and editorials.

## 2. Hydrogels

Hydrogels are three-dimensional (3D) scaffolds composed of networks of hydrophilic polymers crosslinked either through covalent or physical and molecular bonds. Hydrogel classification can be based on source of origin, composition, nature of crosslinking, configuration, ionic charge, properties, and interactions with the environment (chemical and physical responses), as summarized in Table 2 [42,43,44].

### 2.1. Source

Natural polymer hydrogels are intrinsically biocompatible and biodegradable. However, their batch-to-batch variability and diminished chemical stability can complicate experimental reproducibility [45]. Depending on their source, they display different features and tunability. Agarose is a natural, biocompatible, biodegradable, and cost-effective polysaccharide; it forms stable structures via thermal gelation near 37° for slow biodegradation, primarily used for structural stability. Chitosan creates a moist, healing environment and acts as a bioactive carrier. Being alkaline, it requires specific pH or chemical cross-linking for stability. Collagen promotes cell growth and has low immunogenicity. Mechanical strength is often insufficient without chemical blending. Gelatin is a natural, biodegradable material with versatile functional groups (-COOH, -NH_2_) for metal-ion coordination. Its poor thermal stability and mechanical properties require the use of cross-linking agents. Fibrin is a biopolymer that promotes ECM secretion; degradation products are non-toxic. Physically unstable gel state: demanding preparation and processing conditions [43]. Hyaluronic acid is a versatile compound that can be prepared as gels, nano and drug carriers, and hybrid materials, with applications in cartilage regeneration, tissue engineering, and targeted drug release. It can require hybridization with polymers such as PEG for effective binding. Sodium alginate is a natural seaweed-extracted polysaccharide that forms quick physical cross-links with divalent ions (Ca^2+^, Ba^2+^, and Fe^2+^); it is eco-friendly and affordable [43].

Synthetic polymer hydrogels offer absolute molecular precision. Their high customizability enables fine-tuning of stability, permeability, and mechanical properties at an industrial scale, providing controlled biocompatibility and unprecedented application versatility [46]. Polyethylene glycol is a synthetic polymer used to tune physicochemical properties and significantly boost hydrogel strength. Bio-inert: usually requires modification to support cell adhesion. Polyvinyl alcohol (PVA) has high mechanical strength and viscoelasticity; it is non-toxic, non-hazardous, and affordable. PVA is bio-inert, but if blended with natural polymers, it can develop bioactivity [43].

Hybrid hydrogels, combining natural and synthetic biomaterials, can capitalize on the advantages of both by coupling mechanical strength and biocompatibility [47]. For example, an alginate/hyaluronic acid gel that is blue-light-triggered or cross-linked with 1,6-diisocyanate and polyethylene glycol, constitutes an injectable system, showing cartilage regenerative potential [43].

### 2.2. Composition

The molecular composition of the polymeric network determines the hydrogel’s internal architecture and nutrient diffusion. Hydrogels composed of one type of monomer (homopolymers) show uniform swelling behavior [42]. Copolymers incorporating different functional groups allow tuning of hydrogel properties [48].

Advanced compositions include Interpenetrating Polymer Networks (IPNs) and Semi-IPNs. In an IPN, two or more networks are crosslinked but not necessarily covalently bonded, resulting in superior mechanical toughness and multi-stimuli responsiveness. In a Semi-IPN, a linear polymer is physically entrapped within a crosslinked network, which often improves rapid swelling kinetics and provides a mechanism for controlled drug release [49].

### 2.3. Crosslinking

The crosslinking mechanism of a hydrogel dictates the material’s dynamic response and mechanical integrity. These mechanisms are generally categorized by the nature of crosslinking, physical (non-covalent), dynamic covalent, or chemical (permanent), which determines whether the network remains static or can rearrange in response to environmental stimuli [60].

Physical hydrogels are supported by weak, reversible molecular forces such as hydrogen bonding, ionic interactions, and hydrophobic interactions. The networks formed are usually lower in crosslinking density and mechanical strength than those of chemical hydrogels. Nonetheless, they display flexibility and reversibility [50]. An example is alginate hydrogel, which binds divalent ions (such as Ca^2+^) to provide on-demand mechanical tuning for cellular mechanosensing studies. Physiologically, Ca^2+^ ion concentration plays an important role in the function and organization of articular cartilage, as it binds to proteoglycans via electrostatic interactions to maintain the tissue’s structure and function [61].

DCC hydrogels use reversible bonds, which differ from permanent bonds because they are dynamically unstable. They can be divided into four groups based on chemical mechanisms: reversible exchange reactions, reversible addition/condensation reactions, coordinate interactions, and enzymatic/mechanical covalent reactions [51].

Chemical hydrogels have permanent covalent bonds that establish irreversible architectures. This grants them superior thermodynamic stability and a high elastic modulus under mechanical stress [50,52]. These materials are typically static and elastic unless modifications like the photo-initiated polymerization method are made, which provides material robustness, and the enzymatic crosslinking approach, which enables softening or degradation [62].

### 2.4. Configuration

The physical structure and crystalline state of the polymer chains influence the scaffold’s optical clarity and permeability. Amorphous hydrogels are typically transparent and offer high oxygen permeability. Crystalline hydrogels contain regions of highly ordered molecular packing, increasing the elastic modulus and thermal stability but reducing the swelling capacity. Most biomedical hydrogels are Semi-Crystalline, containing a balance of amorphous regions that allow for water absorption and crystalline components that provide resistance to mechanical deformation [63].

### 2.5. Ionic Charge

Neutral network non-ionic hydrogels lack fixed electrical charges. In the context of cartilage tissue engineering, these materials are utilized for their biocompatibility, high water content, and mimicry [54].

Anion and cation hydrogels contain fixed negative or positive charges, respectively, enabling high swelling and smart responsiveness. Anionic hydrogels are helpful for drug delivery, while cationic hydrogels (e.g., chitosan) offer specialized absorption properties. Examples of cations utilized for hydrogels in cartilage tissue engineering are Ca^2+^, Fe^3+^, Mg^2+^, and Zn^2+^ [56]; examples of anions are carboxyl and sulfate groups [55].

Zwitterionic hydrogels have high potential for cartilage tissue engineering due to their ultra-hydrophilicity, lack of immunogenicity, and superior antifouling (preventing the undesirable accumulation of organisms) properties [57].

### 2.6. Properties

The properties of hydrogels define their functional performance within a biological system. Biocompatibility and biodegradability are paramount: hydrogels must support cell adhesion and proliferation while breaking down into non-toxic byproducts. In addition, the swelling ratio is a fundamental property, as it dictates the solute diffusion coefficients and the internal mesh size. This determines how efficiently oxygen, nutrients, and metabolic wastes move through the scaffold. Further, mechanical strength (quantified by Young’s Modulus) must match the stiffness of articular cartilage. Finally, stimulus-sensitivity enables the hydrogel to act to change its physical state in response to the microenvironment, a property that is the cornerstone of modern “smart” biomaterials [58].

### 2.7. Responsiveness

From the perspective of response dynamics, hydrogels can be inert or active (smart).

Traditional hydrogels possess poor resilience, limited self-healing capacity, and static mechanical properties. These restrictions can preclude their use in constantly changing microenvironments.

Stimulus-responsive hydrogels (or “smart hydrogels”) display rapid and reversible phase transitions in response to environmental triggers-such as pH, temperature, or electromagnetic fields-effectively emulating the complexity of living tissues [64].

## 3. Hydrogels for Cartilage Regeneration

### 3.1. Characteristics

Thanks to their high-water content (70–99%) and a structural framework that can mimic the hyaline ECM, hydrogels provide an optimal microenvironment for cell survival and differentiation [65].

Hydrogels offer high flexibility and versatility. They can be produced in various shapes and physical forms, varying their properties at the nanoscopic, microscopic, and macroscopic levels. For example, “smart” hydrogels are stimuli-responsive, or can control the release of growth factors and therapeutic drugs directly at the injury site, thereby actively supporting regeneration and healing [64]. Injectable hydrogels enable administration via minimally invasive techniques, such as arthroscopic injections, and significantly reduce patient morbidity in comparison to traditional open-joint surgeries. Once injected, the hydrogel fluid adapts to irregularly shaped defects, unlike pre-formed scaffolds [66]. Improving implant integration with the surrounding cartilage is associated with better long-term results [67].

The advantages and disadvantages of using hydrogels for cartilage regeneration are summarized in Table 3. The main disadvantages are that natural gels possess high bioactivity but are mechanically fragile, whereas synthetic gels offer structural precision but lack biological characteristics (inertness). The “batch-to-batch variability” of natural polymers is critical since it can alter the immunogenic profile entirely.

### 3.2. Challenges and Strategies

Despite the results achieved in several studies, clinical applications are still lacking. We therefore summarized the problems that emerged from the literature and evaluated the proposed solutions.

**Figure 1:**
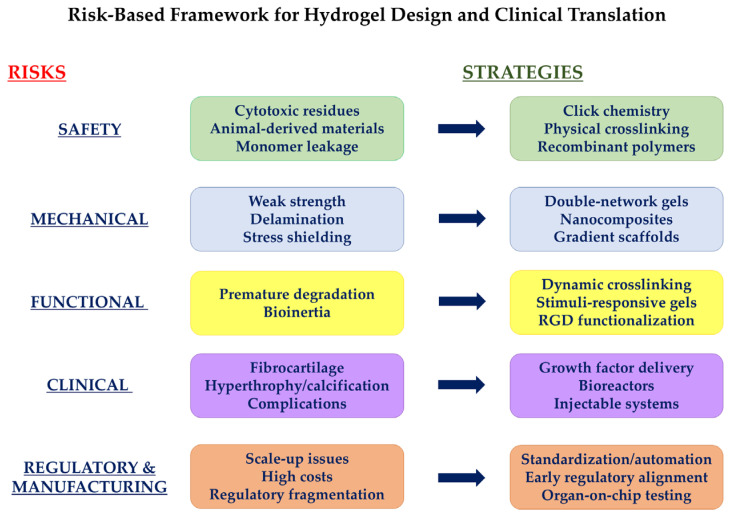
Risk reduction strategies to overcome non-addressed issues in hydrogel-based treatments for the regeneration of articular cartilage. The image was created using Microsoft PowerPoint.

Our search yielded 50 publications, and we picked the ones below this review as exemplifying cases. Based on this, we summarized in Table 4, Table 5, Table 6, Table 7 and Table 8 and Figure 1 the risk reduction strategies that researchers and clinicians are trying to implement to overcome non-addressed issues in hydrogel-based treatments for the regeneration of articular cartilage [6,72].

The prerequisite for the utilization of hydrogel-based scaffolds concerns safety. Chemical cytotoxicity from cross-linking procedures, animal-derived materials, and monomer infiltration [21,48] may cause toxicity, inflammation, immunogenic response, fibrosis, and, eventually, genotoxic effects [49] (Table 4).

Table 4 depicts the need for a complex approach to guarantee patient safety and material performance. Physical crosslinking can reduce chemical toxicity but can also reduce the implant cartilage’s weight-bearing capacity over time. Structural performance is sacrificed in this case to gain safety [77]. Bioorthogonal click chemistry, unlike traditional crosslinking, takes place under physiological conditions with no toxic byproducts, which decreases the possibility of inflammatory responses [73].

The various protocols for producing decellularized ECM improved the de-antigenization process; however, traces of cellular proteins and other non-human epitopes can trigger a chronic “foreign body response,” leading to fibrosis [78]. Current approaches have successfully managed acute toxicity (immediate cell death), but the field still struggles with chronic, low-level irritation. Ultra-rapid in situ gelation that balances hydrogel and drug degradation and is used to develop injectable hydrogels can reduce the window for monomer escape, but it does not account for the long-term degradation of the scaffold [79]. Recombinant human-like proteins, like recombinant human collagen expressed in yeast, could eliminate the risk of animal-origin residues while preserving bioactivity [50,74].

In addition to safety, the clinical success of hydrogel implants depends on their mechanical performance under joint loading. As summarized in Table 5, a major issue for natural hydrogels is their mechanical fragility, as load distribution is compromised [68,80]. Moreover, the risk of delamination and the ‘stress shielding’ effect caused by extremely rigid materials are common causes of implant failure [81,82]. An improvement in mimicking cartilage compressive strength and viscoelasticity is needed to overcome this limitation [83,84].

**Table 5:** The importance of risk assessment and risk reduction strategies for mechanical issues in hydrogel-based constructs.

Risk	Impact	Mitigation Strategy	References
Weak Mechanical Properties	Natural hydrogels lack the strength to withstand high joint loads, leading to an altered load distribution on subchondral bone and accelerating OA development or progression	Hydrogel reinforcement to enhance compressive and shear modulus through nanofillers (e.g., carbon nanotubes, nanocellulose) organic material (i.e., hydroxyapatite) hybridization with synthetic polymers	[68,80]
Delamination and detachment	Failure of integration between the hydrogel and the native cartilage, causing implant failure and freely movable fragments (bodies) in the joint that cause pain	Mollusk catechol modification (inspired by the exceptional underwater adhesion of mussels)	[81]
		Covalent bonds to create injectable, self-healing hydrogels	[82]
		Nanocomposite fillers to restore fluid pressure and seal the surface	[85]
		Design of gradient or bi/three-layer scaffolds to support osteochondral tissue regeneration (with the transitional layer serving as an interface with intermediate stiffness to prevent delamination of the upper and lower layers)	[86]
Stress shielding effect	If the hydrogel is too rigid compared to the surrounding cartilage, it can alter joint load distribution, accelerating osteoarthritis degeneration	Double-network (DN) hydrogels, which combine a sacrificial brittle network to dissipate energy and an elastic one to maintain shape, thus better mimicking cartilage viscoelasticity	[83,84]

The balance between thermodynamic stability (required for joint loading) and reversibility (required for cell remodeling) is a major challenge in hydrogel design and engineering. Stability is guaranteed without deformation under constant load due to permanent covalent bonds. However, they are static, hampering hydrogels from remodeling as cells deposit new ECM [42,63]. Reversible physical bond can dissipate and self-heal through sacrificial bond breaking, but they can also face poor fatigue resistance and excessive stress relaxation for cyclic joint loading [87].

The Double-Network (DN) strategy allows creating tough hydrogels that mimic the resilience of native cartilage [88]. By combining a “sacrificial” first network with a “hidden length” second network, these materials can achieve toughness values greater than those of single-network gels. Network 1 is usually a highly crosslinked, rigid network that dissipates energy by breaking bonds under high stress. Network 2 is a loosely crosslinked, neutral network that maintains the scaffold’s shape and prevents catastrophic crack propagation. Hydrogels with a DN structure that are based on carbohydrates (alginate, cellulose, chitosan, hyaluronic acid, gellan gum, xanthan gum, curdlan gum, and collagen have shown the ability to hold large amounts of water and bioactive molecules [89]. Cai et al. fabricated a hyaluronan-based and gellan gum (HA/GG) double-network (DN) hydrogel that exhibited high compressive strength, stiffness, and self-recovery ability and was able to support chondrocyte proliferation and ECM synthesis in vitro and in vivo (rabbit model) [90].

Recent research has pushed the boundaries of the DN framework to a material that mimics both boundary and biphasic lubrication mechanisms of cartilage. A poly(2-methacryloyloxyethyl phosphorylcholine) (PMPC) polymeric network was incorporated into a DN biphasic) gel to form a PMPC triple-network (PMPC TN) hydrogel that exhibited boundary and lubrication capability, and a yield stress of 26 MPa, which is an order of magnitude higher than the peak stresses found in the native human knee. The use of PMPC zwitterionic polymer, which maintains high hydration and lubricity, improved the frictional properties of the tough DN part [91].

Optimizing the cross-linking process and developing stimulus-reactive materials that degrade specifically in response to specific stimuli can be a strategy [74,92]. Stimuli-reactive degradation is promising, but there are still problems to fix. Most “smart” hydrogels respond to pH or temperature; however, in a damaged joint, stimuli arise from degradation/inflammatory/fibrotic factors, such as matrix metalloproteinases (MMPs), reactive Oxygen Species (ROS), and mechanical stress. The OA microenvironment is characterized by the overexpression of matrix metalloproteinases (MMPs) and by mechanical stimuli from joint movement. A dual-responsive injectable hydrogel, including a blend of MMP-responsive, thermo-sensitive Gelatin Methacryloyl (GelMA) and a mechanically robust, reverse thermo-sensitive F127 Diacrylate (F127DA) hydrogel micelles, was designed to deliver TGF-β (critical regulator of cartilage homeostasis) and KGN (promotes the proliferation and chondrogenic differentiation of MSCs) in a controlled manner via temperature modulation. GelMA has hydrophilic properties and is degraded by MMPs that recognize and cleave peptide bonds. However, as a delivery vector, GelMA ignores the joint’s mechanical loading microenvironment. F127DA has a hydrophobic nature, and its self-assembled nano-micelles can load the small-molecule hydrophobic drug KGN, serving at the same time as crosslinking centers, providing an additional energy dissipation mechanism. The dual-network GelMA-F127DA hydrogel (GF hydrogel) system was able to respond to the overexpressed MMPs in the OA environment, triggering the release of TGF-β, recruiting bone marrow-derived stem cells (BMSCs), while mechanical pressure from joint movements releases KGN, promoting chondrogenic differentiation and mitigating inflammation [93].

Mechanically robust synthetic hydrogels are generally bioinert and can be functionalized by several strategies [92]. For example, Arginine-Glycine-Aspartic acid (RGD) can promote cell adhesion and viability and prevent fibrosis. By providing specific integrin-binding sites, RGD-modified hydrogels create a biomimetic environment that encourages the chondrogenic phenotype rather than a fibrotic one [94].

**Table 6:** Functional issues in hydrogel-based systems: risk assessment and risk reduction strategies.

Risk	Impact	Mitigation Strategy	References
Premature material degradation	Material degrades before new cartilage formation. If reabsorption is faster than matrix production, the structural support collapses, leading to intervention failure.	Optimization of cross-linking: dynamic bonds, such as disulfide bonds, with self-healing properties that allow the hydrogel to autonomously reform after damage	[92,95]
		Development of stimulus-reactive materials	
Bioinertia	Synthetic hydrogels, although mechanically strong, may not offer the biological signals necessary for cell adhesion, migration, and proliferation.	Functionalization with RGD peptides or growth factors (TGF-β)	[92]
		Blending with natural polymers (collagen, hyaluronic acid) to provide intrinsic biocompatibility	

The basis for the clinical success of hydrogel-based treatments is tissue integration, neo-tissue formation, and long-term biological stability. As synthesized in Table 7, one of the main risks is the formation of fibrocartilage rather than functional hyaline tissue. Utilized strategies include the controlled release of chondrogenic factors (for instance, TGF-β) [80,92], the creation of a proper microenvironment by the incorporation of adhesive sequences [81,96], and the use of bioreactors to simulate the joint through dynamic compression cultures before implantation [95]. Furthermore, porosity modulation through 3D-printed scaffolds can support cell viability and upregulate key hyaline markers, like collagen type II [97].

The risks of hypertrophic differentiation and calcification caused by high crosslinking densities can be prevented by lowering the density. For instance, low-molecular-weight methacrylated hyaluronic acid (MeHA) can promote a favorable microenvironment for hyaline regeneration [98,99].

Injectable hydrogels and microneedles substantially reduce treatment invasiveness and post-operative complications by precise filling [66].

**Table 7:** Clinical issues in hydrogel-based systems: risk assessment and risk reduction strategies.

Risk	Impact	Mitigation Strategy	References
Formation of fibrocartilage	The neo-formation of fibrocartilage, which has lower mechanical properties and tends to degrade rapidly, at the expense of naïve hyaline cartilage	Controlled release of growth factors: development of controlled release hydrogels. For example, the growth factor TGF-β drives the differentiation of stem cells towards the hyaline chondrocyte phenotype.	[80,92]
		Modifying polymeric hydrogel chains with adhesive peptide sequences, most notably RGD.	[81,96]
		Porosity modulation: human bone marrow-derived MSC-based spheroids associated with 3D porous PEGDA hydrogels supported the viability and chondrogenic differentiation, as demonstrated by the positive staining of the ECM for glycosaminoglycans and upregulation of chondrogenesis marker genes, like collagen type II.	[97]
		Pre-implantation Bioreactors: Before clinical use, apply cell-seeded hydrogel to dynamic compression cycles in specific bioreactors to “train” the cells to produce hyaline ECM.	[95]
Hypertrophic chondrocytes differentiation and ECM calcification	Hyaluronic acid-based hydrogels with a high crosslinking density, preventing the migration of nutrients, can promote the production of a calcified ECM, leading chondrogenic-stimulated MSCs to differentiate into a hypertrophic phenotype (expressing type X collagen)	Low-density or low-molecular-weight hydrogels: methacrylated hyaluronic acid (MeHA) can protect and promote the formation of articular cartilage. They achieve this by improving integration into the cartilage ECM, reducing degradation, and providing a favorable microenvironment for proper tissue regeneration.	[98,99]
Invasive treatment	Patient discomfort during treatment	Development of minimally invasive options: hydrogel microneedles.	[66]
Post-operative complications	Risks of infection or joint stiffness	Injectable hydrogels allow for the treatment of irregularly shaped defects.	[66]
		Follow-up monitoring: for example, the use of magnetic resonance imaging with the Magnetic Resonance Observation of Cartilage Repair Tissue (MOCART) score to detect pilot release or filling abnormalities.	[100]
		Design control: Use of computational models to predict the behavior of the hydrogel under load before implantation.	[63]
Long-term uncertainty	Most clinical trials on hydrogel-based applications for cartilage regeneration are still in the initial stages, which means that long-term safety data (over 10 years) is still being consolidated	Solid clinical data on the duration of results beyond 5–10 years are still lacking.	[101]

The limited long-term data on the safety and efficacy of hydrogel-based strategies for the treatment of cartilage defects identifies a bottleneck for this kind of therapy [63,100,101].

Without long-term data, it is not possible to confirm the durability of the outcomes. This gap largely depends on cartilage regeneration, a prolonged, complex, and sometimes discontinuous process [102]. Hypertrophic differentiation or fibrous degeneration may escape short-term follow-ups and manifest gradually over time. Additionally, the heterogeneity introduced by hydrogel types and compositions, cell types and manipulations, and cartilage treatment approaches is an obstacle to evidence-based design trials. Thirdly, early-phase trials, for instance, early-onset OA trials, enroll young, healthy participants. Thus, data are missing for older patients with metabolic comorbidities or systemic inflammation.

Another factor hampering long-term outcomes trials is the scarcity of standardized, reproducible assessments of regenerated cartilage tissue. An increasing number of surgical techniques and available scaffolds have been developed since the introduction of ACI [7]. Consequently, follow-up monitoring has become as well important.

Clinical scores, such as the Lysholm Knee Score [103], the IKDC (International Knee Documentation Committee) score [104], or the KOOS (Knee Injury and Osteoarthritis Outcome Score) score [105], reflect the individual disease burden and overall joint health. However, these scores lack specificity regarding the quality and state of the repair tissue itself. MRI allows a non-invasive measurement of proteoglycan content and collagen fiber orientation for the morphological assessment of cartilage defects and maturing repair tissue throughout the post-operative period. However, morphological MRI is based on qualitative assessment and thus suffers from a fundamental lack of standardization and objectivity. The Magnetic Resonance Observation of Cartilage Repair Tissue (MOCART) is a semi-quantitative score based on nine variables for the morphological assessment of cartilage repair [106]. Quantitative MRI allows for more precise monitoring by the quantification of tissue physical properties. Spin–spin relaxation time (T2) and spin–lattice relaxation time constant in rotating frame (T1rho) mapping, the most studied cartilage biomarkers, were included in the recent standardization effort by the Quantitative Imaging Biomarkers Alliance (QIBA) that aims to make compositional MRI of cartilage clinically feasible and comparable [107]. Additional techniques that are less frequently used include Delayed Gadolinium-Enhanced MRI of Cartilage (dGEMRIC), which assesses cartilage health by measuring glycosaminoglycan (GAG) content, enabling visualization of early cartilage degeneration, especially in knee and hip joints [100]; glycosaminoglycan concentration by chemical exchange-dependent saturation transfer (gagCEST), sodium imaging, and diffusion-weighted MRI [107].

Predictive systems can be helpful in the design of clinical trials, like in silico Stress Modeling using patient-specific joint-loading data to simulate many years of “wear and tear” [108]. Other predictive biomarkers under investigation are specific pro-inflammatory cytokines present in the knee, present in OA synovial fluid at different concentrations during follow-up.

Table 8 describes how the regulatory panorama fragmentation and classification ambiguity may create a regulatory limbo. For example, exosomes that can carry proteins or genetic material, or a 3D-bioprinted tissue, are Advanced Therapy Medicinal Products (ATMPs), medical devices (MDs), or biological transplants? International standards, strategic harmonization, and early, frequent consultation with regulatory bodies are essential. Early consultation with regulatory experts is cardinal for achieving global market harmonization [109,110]. Consolidated regulatory and manufacturing requirements should control the transition of next-generation hydrogels from laboratory to clinical practice [109,110].

The development of 3D bio-printed and organ-on-a-chip human models is not only a response to animal rights concerns but also stems from the observation that animal models often fail to accurately predict human systemic changes.

Moreover, the process complexity and high production costs demand a shift toward standardized, automated manufacturing to ensure scalability [111]. The industry is dealing with the challenge of long R&D timelines through milestone-based pilot studies to validate data early in the development cycle [112]. It is also important to consider the scalability of a hydrogel system and its clinical applicability. Does the treatment want to be selective or available in as many hospitals as possible? Those are considerations to make in the very early phases of R&D.

Finally, the growing ethical scrutiny on animal testing and the need to prevent costly biocompatibility failures urged researchers to adopt alternatives such as advanced in vitro technologies, like organ-on-a-chip models, which provide better predictability than standard methods [113] and 3D bioprinted human tissue models able to reproduce the 3D articular environment [114].

**Table 8:** Regulatory and manufacturing issues in hydrogel-based systems: risk assessment and risk reduction strategies.

Risk	Mitigation Strategies	Reference
Long R&D complications: the R&D phase is complex, multi-faceted, multi-step, costly, long, and time-consuming	Milestone-based approach: pilot studies and targeted pre-clinical testing to validate data before investing in large-scale clinical trials	[112]
Complex and expensive production process: (for instance, nanocomposites, organ-on-a-chip)	Scalability and standardization: optimizing manufacturing processes and investing in automation	[111]
Biocompatibility failures: ISO 10993 ensures device safety for human use by evaluating biocompatibility	Advanced in vitro testing: organ-on-a-chip models to test biocompatibility, avoiding failures during mandatory in vivo testing	[113]
Ethical and animal testing barriers: without animal testing, researchers may fail to see joint and systemic changes	Adoption of validated alternative methods, such as 3D bioprinted human tissue models	[115]
Regulatory compliance issues: lack of communication with regulatory bodies like the FDA (USA) or EU-designated authorities	Early regulatory consultation: contact regulatory bodies or engage regulatory affairs experts just at the initial design phase	[109]
Regulatory panorama fragmentation: regulations vary, which impedes manufacturers from selling products across multiple countries	Strategic harmonization: international standards (like ISO 13485) and guidance to facilitate mutual recognition across global markets	[110]
Unclear classification of exosomes: Exosomes may be classified as drugs, biological products, or ATMPs depending on the region	Meticulous characterization: Define chemistry, manufacturing, and control (CMC) requirements early to align with biological medicinal product standards	[116]
Unclear classification of 3D-bioprinting products: Ambiguity between classification as medical devices, biologics, or Advanced Therapy Medicinal Products (ATMPs)	Case-by-case classification: Early engagement with regulatory bodies to determine the primary mode of action and appropriate pathway	[117]
Lack of organ-on-a-chip standardization: Design variability leads to poor data reproducibility and skepticism	Unified Roadmap: Adopt standardized protocols for chip manufacturing and cell differentiation to ensure data robustness.	[118]

## 4. Future Perspectives

This review addresses the significant challenges involved in translating hydrogel-based systems from the laboratory to clinical application and defines mitigation strategies for cartilage regeneration.

Based on literature and our experience, future perspectives would focus on protocol standardization, rigorous quality control, and scalable manufacturing to ensure reproducibility. Consultation with regulatory authorities is essential for product classification. To validate outcome data, long-term clinical trials are necessary, and integrating predictive models, such as organ-on-a-chip technologies, can further aid clinical translation.

## Figures and Tables

**Table 2:** Overview of hydrogel classification.

Classification	Type	Characteristics	Refs.
Source	Natural	Derived from biological sources (e.g., collagen, chitosan, alginate)	[45]
	Synthetic	Synthesized using chemical polymerization (e.g., PEG, PAA)	[46]
	Semi-synthetic	Natural polymers modified with synthetic groups to enhance properties	[47]
Composition	Homopolymer	Consists of only one type of monomer in the network	[42]
	Copolymer	Formed by two or more different types of monomers	[48]
	Semi-IPN	One crosslinked network with a second “linear” polymer trapped inside	[49]
	IPN	Two or more independent, cross-linked networks interlaced at a molecular level	
Crosslinking	Physical junction	Reversible bonds (hydrogen bonds, ionic interactions, or chain entanglements)	[50]
	Chemical binding	Permanent, stable covalent bonds between polymer chains	[51,52]
Configuration	Amorphous	Disordered, random molecular arrangement (transparent and flexible)	[53]
	Crystalline	Highly ordered, tight molecular packing (stronger and opaquer)	
	Semi-Crystalline	A mixture of both ordered crystalline and disordered amorphous regions	
Ionic Charge	Nonionic	Neutral network with no electrical charge	[54]
	Anionic	Carries a negative charge (often responds to higher pH)	[55]
	Cationic	Carries a positive charge (often responds to lower pH)	[56]
	Ampholytic	Contains both positive and negative charges (zwitterionic)	[57]
Property	Mechanical strength	The ability of the gel to withstand physical stress or load	[58]
	Biocompatibility	Compatibility with living tissues without causing harm	
	Biodegradability	Capacity to break down naturally in a biological environment	
	Swelling ability	The ability to absorb and hold large amounts of water or fluids	
	Stimuli sensitivity	Ability to change volume or shape in response to environmental cues	
Chemical Response	Stimuli Factors	Responds to pH, ionic strength, solvent composition, molecular species, and redox reactions	[59]
Physical Response	Stimuli Factors	Responds to temperature, electric field, magnetic field, light, pressure, sound, and humidity	

**Table 3:** The advantages and disadvantages of using hydrogels in cartilage regeneration.

Advantage	Disadvantage	
Biomimicry: high water content and gel structure resembling cartilage ECM support cell survival, proliferation, and differentiation	Low mechanical strength: lack of compressive and shear strength required to withstand the high-load joint environment	[43]
Biocompatibility: natural-based hydrogels (hyaluronic acid, collagen, or chitosan) have a minimal risk of adverse or toxic reactions	Batch variability: natural polymers can suffer from inconsistent properties between batches, affecting reproducibility in clinical settings	[68]
Non-immunogenicity: the ability to trigger an appropriate host response without causing an inappropriate immune response	Degradation-induced inflammation: some synthetic components or cross-linking agents may trigger a delayed foreign-body response during degradation	[69]
Biodegradability: controlled breakdown of the material into non-toxic byproducts	Mismatch in rates: Difficulty in matching the hydrogel degradation rate with the speed of new tissue formation, potentially leading to premature structural failure	[43]
Design Flexibility: hydrogels can be engineered to be bioadhesive, biodegradable, or to actively release growth factors and drugs to promote healing	Complex release kinetics: controlling the burst effect or maintaining a sustained release of bioactive factors over long periods is technically difficult	[70]
Customizability: patient-tailored, stimuli-responsive, or “smart” behavior hydrogels can be engineered from the molecular level (composition and cross-linking) to macroscopic architecture (shape and responsiveness)	Technical complexity: high manufacturing costs and complex regulatory pathways for “smart” or highly engineered materials	[71]
Minimal invasiveness: hydrogels can be injected directly into the joint, allowing for precise, arthroscopic, minimally invasive surgery rather than open joint surgery	Leakage and migration risk: low-viscosity precursor solutions may leak from the defect site before gelation (cross-linking) is complete	[66]
Effective for focal defects: hydrogels can fill irregularly shaped cartilage voids, providing a better fit and structural support for a specific injury	Poor lateral integration: difficulty in achieving a seamless biological and mechanical bond between the hydrogel and the surrounding native cartilage	[66]

**Table 4:** Residue presence in hydrogel-based constructs: risk assessment and risk reduction strategies.

Risk	Impact	Mitigation Strategy	References
Cytotoxic chemical residues from the crosslinking process	Toxic or inflammatory responses leading to fibrocartilage formation	Physical crosslinking (UV/temperature) or Bio-orthogonal click chemistry to eliminate toxic catalysts	[73]
Animal origin residues	Rejection, allergic reactions, cartilage hypertrophy, and fibrosis	De-antigenization protocols for natural polymers or the use of ultra-pure synthetic precursors	[50,74,75]
Leaking of monomers (e.g., acrylic acid, acrylamide)	Cytotoxic and genotoxic effects	Controlled degradation	[76]

## Data Availability

No new data were created or analyzed in this study. Data sharing is not applicable to this article.
